# Are “additional cuts” effective for positive margins in cervical conization? It varies according to the doctor

**DOI:** 10.1186/s12957-023-03119-1

**Published:** 2023-08-23

**Authors:** Yujie Sun, Yingying He, Mima Zhuoma, Zhengyu Hua, Zhigang Sun, Nan Jiang, Fandou Kong, Zhen Xiao

**Affiliations:** 1 Department of Obstetrics and Gynecology, First Affiliated Hospital of Dalian Medical University, Dalian, China; 2 Graduate School of Dalian Medical University, Dalian, China; 3 Department of Pathology, People’s Hospital of Pingshan District, Shenzhen, China; 4 Institute of High Altitude Medicine, People’s Hospital of Naqu Affiliated to Dalian Medical University, Dalian, Tibet, China; 5 Department of Pathology, First Affiliated Hospital of Dalian Medical University, Dalian, China

**Keywords:** Additional cuts, Cone depth, Cone volume, Doctor’s habit, High-grade squamous intraepithelial lesion, Cervical cancer

## Abstract

**Background:**

High-grade squamous intraepithelial lesion (HSIL) is a disease that is closely related to the development of cervical cancer. In clinical work, cold knife conization and a loop electrosurgical excision procedure (LEEP) are often selected for diagnosis and treatment.

**Objective:**

In this paper, we aimed to discuss additional cuts, a common practice in cervical conization, and determine whether the doctor’s choice to use additional cuts in conization can reduce the occurrence of a positive cone margin.

**Methods:**

From January 2018 to October 2019, 965 patients underwent cervical conization at the First Affiliated Hospital of Dalian Medical University (Dalian, China). Of these, 174 were in the positive cone margin group, and 791 were in the negative cone margin group. Age, preoperative pathology, pathological results of conization, additional cuts, cone depth, and cone volume were studied. Additionally, the additional cut rate and the efficiency of doctors with a habit of additional cuts were analyzed.

**Results:**

Of the 965 patients included in the study, the median age was 41 years (range 35–50). Multivariable logistic regression analysis suggested that additional cuts (*OR*, 2.480; 95% *CI* 1.608 to 3.826; *p* = 0.01) and smaller cone depth (*OR*, 0.591; 95% *CI*, 0.362 to 0.965, *p* = 0.036) were independent risk factors for positive margins. Six of the 64 doctors who performed conizations had a habit of making additional cuts, and there was no positive correlation between their additional cut rate and their effective additional cut rate.

**Conclusion:**

This study showed that a certain proportion of additional cuts can be effectively excised from the positive margin that cannot be removed in the initial conization. The practice of additional cuts in conization tends to be the personal habit of a small number of doctors.

**Graphical Abstract:**

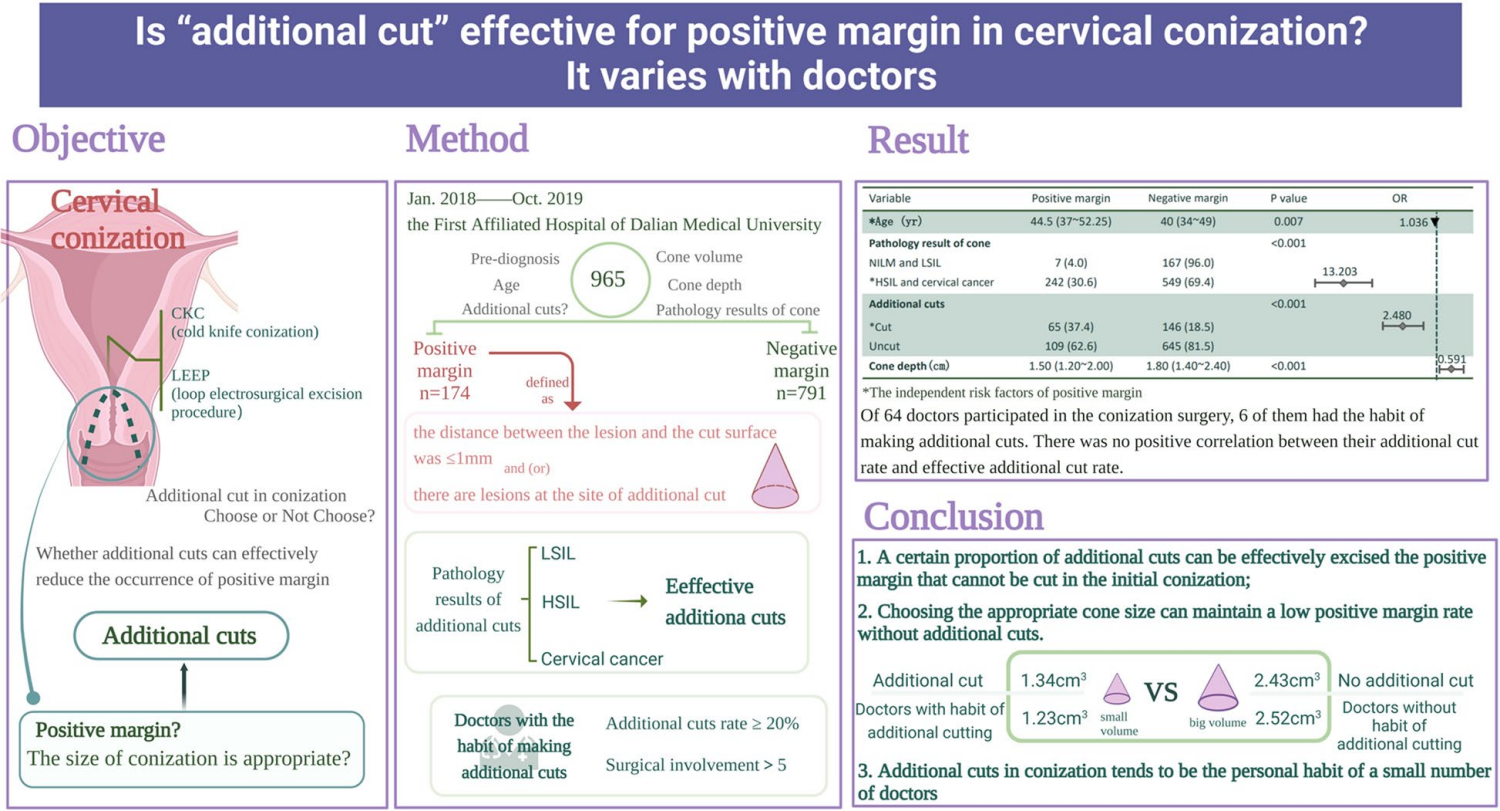

## Background

Cervical cancer is one of the most common cancers among women, and the mortality of cervical cancer is high. In 2020, there were an estimated 604,000 new cases and 342,000 deaths worldwide. Research suggests that squamous intraepithelial lesions (SILs) caused by persistent infection with human papillomavirus (HPV) are closely related to the occurrence of cervical cancer, so effective screening measures for SILs can be helpful in preventing this condition [1, 2]. For women with histologically confirmed high-grade squamous intraepithelial lesions (HSILs), cold knife conization (CKC), a loop electrosurgical excision procedure (LEEP; including large loop excision of the transformation zone or cone biopsy with loop excision), and laser conization (LC) are typically used for diagnostic purposes and as a principal treatment approach [3].

In clinical work, it is common to have positive margins after cervical conization. A meta-analysis showed that approximately 25% of patients experience incomplete excision, and some researchers consider the proportion of patients with complete excision of lesions to be a quality criterion for clinical practice [4]. Several variables, such as age more than 50 years, high parity, and menopausal status, have been reported to be associated with positive margins [5, 6]. Furthermore, a positive margin is one of the main causes of HSIL recurrence. For women with a positive margin, there is a higher risk of residual or recurrent HSIL or worsening disease than for women with a clean margin, and patients with a positive cone margin had a nearly 2.7-fold recurrence rate compared to patients with a negative cone margin [5, 7, 8]. Consequently, when performing conization surgery, some doctors may worry about the positive margin shown on the pathology reports and whether their cut range is not sufficient, and they choose to make additional cuts when performing conization. However, whether additional cuts can effectively avoid the occurrence of positive margins is still unclear. Only a few articles have mentioned the use of additional cuts when necessary, and additional cuts do not appear to have a good preventive effect on cervical cancer [9, 10]. Additionally, to the author’s knowledge, there is little information in the literature about the association between positive margins and the choice to make additional cuts.

In this study, we retrospectively analyzed pathology report data from patients who underwent cervical conization to determine whether making additional cuts can reduce the rate of positive margins in cervical conization. This study aimed to provide suitable evidence to help doctors choose whether to make additional cuts in cervical conization.

## Methods

**Fig. 1:**
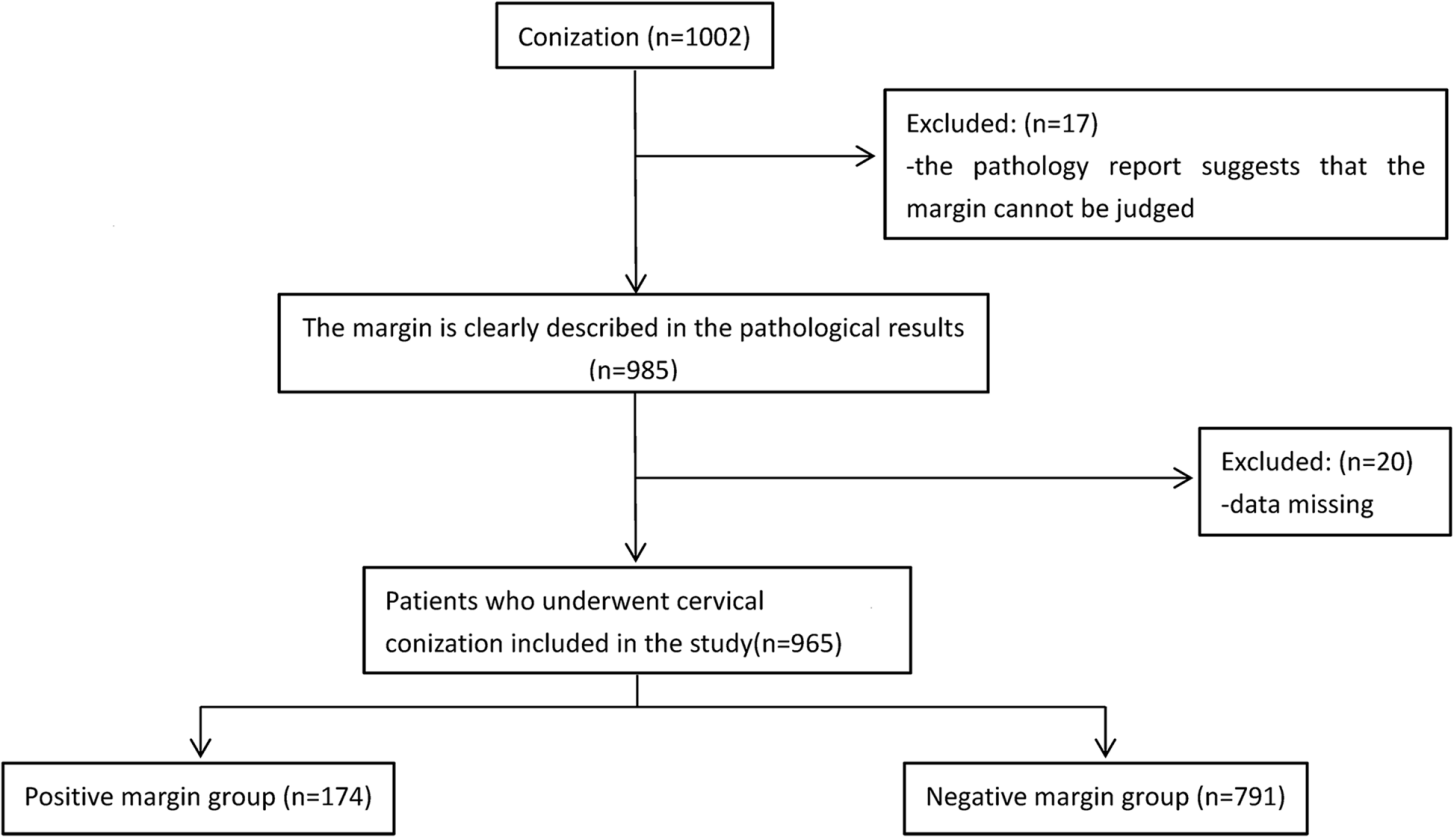
Patient selection flowchart. Legend: After excluding 37 patients, 965 patients who underwent cervical conization out of 1002 patients were finally selected for inclusion in this study. According to the conization results, there were 174 patients in the positive margin group and 791 patients in the negative margin group

We retrospectively reviewed the records of 1002 patients who underwent cervical conization (including CKC and LEEP) at the First Affiliated Hospital of Dalian Medical University (Dalian, China) from January 2018 to October 2019, including patients with a preoperative diagnosis of HSILs, cervical squamous carcinoma, adenocarcinoma in situ (AIS), and low-grade squamous intraepithelial lesions (LSILs). Of the 1002 patients, 965 patients had clear margin results on their pathology reports and complete data, excluding 17 patients with cut margins that could not be assessed and 20 patients with missing data (Fig. 1). According to the pathological results of the conization, the patients were divided into two groups: a positive cone margin group (*n* = 174) and a negative cone margin group (*n* = 791). This study was approved by the Ethics Committee of the First Hospital of Dalian Medical University.

In previous studies of conization, positive margins were defined as lesions (LSIL, HSIL, or cervical cancer) at or near ($\le$ 1 mm) the cut surface, [11, 12] but in this study, the definition of positive margins included a distance between the lesion and the cut surface of $\le$ 1 mm and/or lesions at the site of an additional cut. This expansion of the definition of positive margins allowed unclean margin cases, such as margins that were negative but had lesions at the additional cut site, to be included in the study. For patients who underwent additional cuts, the additional cut was determined to be effective if there was a lesion (LSIL, HSIL, or cervical cancer) at the site of the additional cut. In this study, we defined doctors who have the habit of making additional cuts as those with an additional cut rate greater than 20% who have performed more than five surgeries.

IBM SPSS statistics version 25 was used for statistical analysis. The chi-square test (${\chi }^{2}$ test) and Mann-Whitney U test were used to compare clinicopathological variables (age, preoperative pathology results, pathology results of conization tissue, additional cuts, and cone depth and volume) between the positive margin group and the negative margin group. Multivariable logistic regression analyses were used to test the value of clinical parameters in predicting positive margins. A *p*-value of $<$ 0.05 was considered statistically significant.

## Results

The patient characteristics are presented in Table 1. Of the 965 study patients, the median age was 41 years (range 35–50). According to the pathology results for diagnosis before conization, 2.5% had LSILs, and 97.5% had HSILs or cervical cancer. Only a small fraction (3.7%) of the pathological results of the conization tissue were negative for intraepithelial lesions or malignancy (NILM); HSILs and cervical cancer accounted for the majority (74.2%). The median cone depth and volume were 1.80 cm (range 1.30–2.30) and 1.81 cm^3^ (range 1.18–2.94), respectively.

**Table 1:** Characteristics of 965 patients

Characteristics	Values
**Age (year)**	
Median	41
25th–75th percentile	35–50
≤ 40	467 (48.4)
> 40	498 (51.6)
**Pathology result (pre-diagnosis)**	
LSIL	24 (2.5)
HSIL	915 (94.8)
Cervical cancer	26 (2.7)
**Pathology result of conization tissue**	
NILM	36 (3.7)
LSIL	213 (22.1)
HSIL	665 (68.9)
Cervical cancer	51 (5.3)
**Cone depth (cm)**	
Median	1.80
25th–75th percentile	1.30–2.30
**Cone volume (cm**^**3**^**)**	
Median	1.81
25th–75th percentile	1.18–2.94

Values are presented as median (interquartile range) or number (%). *LSIL*, low-grade squamous intraepithelial; *HSIL*, high-grade squamous intraepithelial; *NILM*, negative for intraepithelial lesions or malignancy

We evaluated the correlation between patient characteristics and cone margin status, and we used the Mann-Whitney *U*-test and chi-square test to evaluate the association between factors and positive margins (Table 2). Older age (*p* = 0.007), a pathology result of conization tissue of HSILs or cervical cancer (*p* $<$ 0.001), choosing to make additional cuts (*p* $<$ 0.001), smaller cone depth (*p* $<$ 0.001), and smaller cone volume (*p* = 0.01) had significantly higher rates of positive margins in the overall cohort.

**Table 2:** Correlation between factors and cone margin status

Variable	Positive margin cone group	Negative margin cone group	*p*-value
Age (year)*	44.5 (37–52.25)	40 (34–49)	0.007
Pathology result (pre-diagnosis)*			0.074
LSIL	1 (0.6)	23 (2.9)	
HSIL and cervical cancer	173 (99.4)	768 (97.1)	
Pathology result of conization tissue*			< 0.001
NILM and LSIL	7 (4.0)	242 (30.6)	
HSIL and cervical cancer	167 (96.0)	549 (69.4)	
Whether to do additional cuts**			< 0.001
Cut	65 (37.4)	146 (18.5)	
Uncut	109 (62.6)	645 (81.5)	
Cone depth (cm)*	1.50 (1.20–2.00)	1.80 (1.40–2.40)	< 0.001
**Cone volume (cm**^**3**^)*	1.57 (1.17–2.26)	1.88 (1.18–3.01)	0.01

Values are presented as median (interquartile range) or number (%)

*LSIL* low-grade squamous intraepithelial, *HSIL* high-grade squamous intraepithelial, *NILM* negative for intraepithelial lesions or malignancy, *AC* additional cut

^*^p-valve by Mann–Whitney *U*-test

^**^p-value by chi-square test

**Fig. 2:**
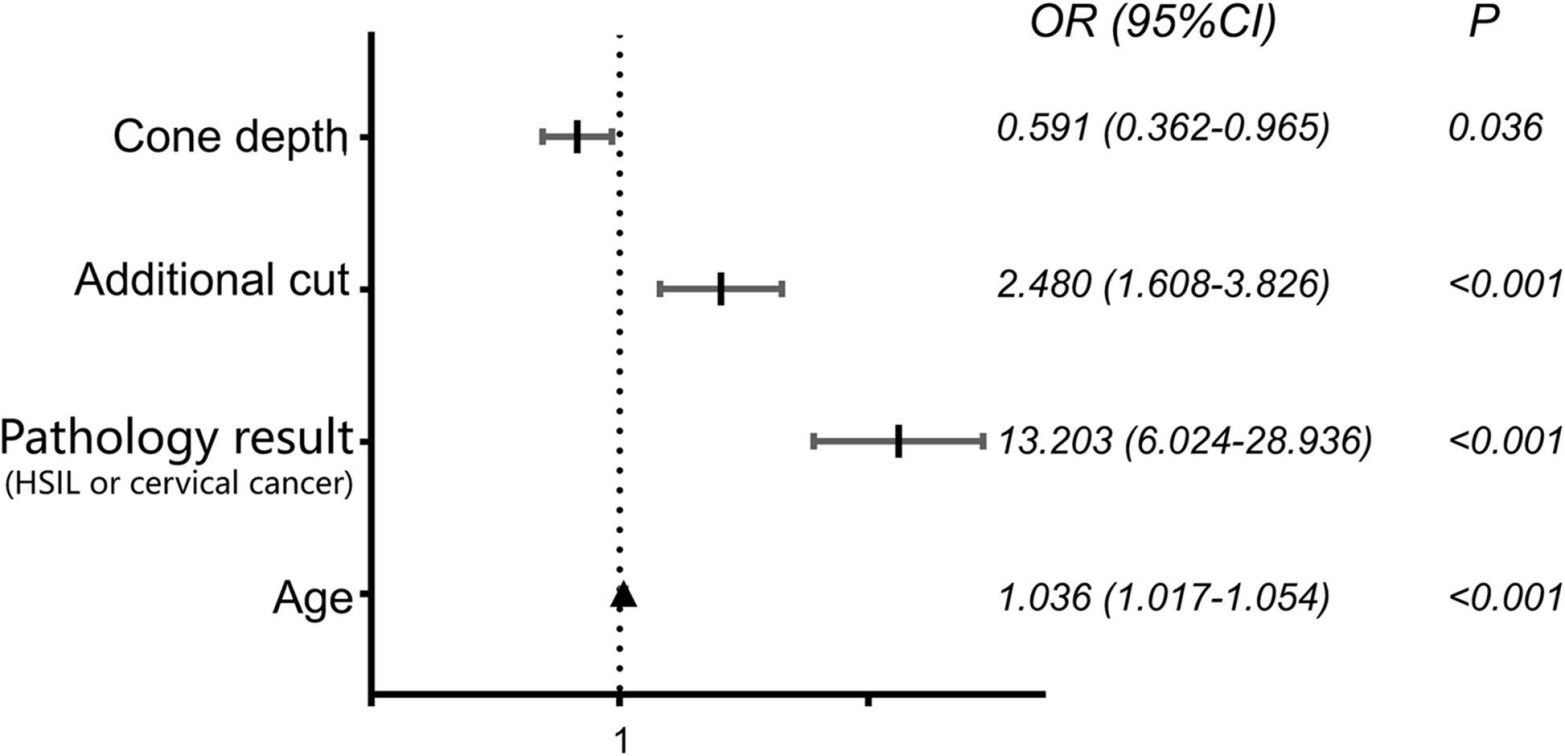
Multivariable logistic regression analysis of the correlation between clinicopathological factors and positive margins. Legend: In cervical conization, older age, shorter cone depth, additional cuts, and pathology result of conization tissue of HSILs or cervical cancer were independent risk factors for positive margins in conization

Multivariable logistic regression analysis revealed that making additional cuts (odds ratio [OR], 2.480; 95% *CI* 1.608 to 3.826; *p* = 0.01), a pathology result of conization tissue of HSILs or cervical cancer (*OR*, 13.203; 95% *CI*, 6.024 to 28.936; *p* $<$ 0.001), age (*OR*, 1.036; 95% *CI*, 1.017 to 1.054; *p* $<$ 0.001), and smaller cone depth (*OR*, 0.591; 95% *CI*, 0.362 to 0.965, *p* = 0.036) were independent risk factors for positive margins (Fig. 2).

Subsequently, we analyzed the additional cut rate and the effective additional cut rate of doctors who had a habit of making additional cuts (Table 3). In our study, a total of 64 doctors performed conization surgeries, six of whom (9.4%) had a habit of making additional cuts; four of these doctors had an additional cut rate greater than 80%. Of these six doctors, one doctor’s additional cuts were ineffective, and the rest of the doctors had a relatively high effective additional cut rate.

**Table 3:** Doctor who has the habit of making additional cuts

Doctor	Amount of surgical involvement	Additional cuts rate (%)	Effective additional cuts rate (%)
A	82	34.1	25.0
B	57	98.2	33.9
C	49	95.9	10.6
D	35	88.6	22.6
E	8	87.5	14.3
F	13	30.8	0.0

Values are presented as number or percentage (%)

Table 4 shows that the cone volume in the additional cuts group ranged from 0.89 to 1.57 cm^3^ (median = 1.25 cm^3^), and there was an extremely significant difference (*p* $<$ 0.001) in cone volume between the group without additional cuts and the group with additional cuts. Doctors who had a habit of making additional cuts had significantly smaller cone volumes than doctors who did not have this habit (median = 1.16 cm^3^ and 2.20 cm^3^, respectively; *p* $<$ 0.001).

**Table 4:** Correlation between factors and cone volume

Variable	Cone volume (cm^3^)	*p*-value
**Whether to do additional cuts**		< 0.001
Yes (*n* = 211)	1.25 (0.89–1.57)	
No (*n* = 754)	2.09 (1.31–3.27)	
**Doctors’ habit**		< 0.001
Like making additional cuts (*n* = 252)	1.16 (0.87–1.47)	
Do not like making additional cuts (*n* = 713)	2.20 (1.43–3.27)	

Values of volume are presented as median (interquartile range). *p*-value by Mann–Whitney *U*-test

## Discussion

### Main findings

In this study, we observed that older age, shorter cone depth, making additional cuts, and a pathology result of conization tissue of HSILs or cervical cancer were independent risk factors for positive margins in conization. Additional cuts were determined to be an independent risk factor for a positive surgical margin because in the included cases, most patients with a positive margin underwent additional cuts. Among doctors who have a habit of making additional cuts, the cuts were overwhelmingly effective. Additionally, the cone volume was significantly smaller in cases with additional cuts and in surgeries performed by doctors who had a habit of making additional cuts.

### Strengths and limitations

A number of studies have analyzed the influencing factors associated with positive cone margins. However, to our knowledge, this study is the first study on the problem of making additional cuts in cervical conization.

There are limitations of this study. This analysis included only 965 pathology reports at the First Affiliated Hospital of Dalian Medical University (Dalian, China), and only 64 doctors were involved. Moreover, there are few studies on additional cuts in cervical conization, resulting in a lack of research in other hospitals or regions, and the generalizability of the results is uncertain. We cannot definitively say that additional cuts can reduce the occurrence of positive margins in conization. A retrospective Korean study of 65 cases showed that conization type and cone volume were statistically significant factors in preterm delivery [13], but we were unable to investigate whether additional cuts had an impact on preterm delivery due to a lack of follow-up of patients.

### Interpretation

Chinese practices for cervical conization [14] recommend that the indication for cervical conization surgery is cervical cytology of HSIL, AIS, or cervical cancer. Additionally, in clinical practice, patients who underwent conization surgery mainly had HSILs. However, in this experiment, 2.5% of patients had a prediagnosis of LSILs (Table 1), largely because doctors believe that those patients had the potential to develop the disease, had different grades of lesions at the biopsy site, or were missed or misdiagnosed cases of HSILs. This was similar to a Japanese report [15] on the prediagnosis of cervical intraepithelial neoplasia (CIN) 1 and 2 in patients with conization. Approximately, half of patients initially diagnosed with CIN 1 and 2 actually had CIN 3 or invasive cancer in the cervical tissue. Therefore, it is necessary to use actual clinical observations to decide whether to perform conization in patients with LSILs.

In cervical conization, a positive margin was strongly associated with the persistence and recurrence of HPV, and a positive margin was a major risk factor for predicting the 5-year recurrence rate. Positive margins were also important for prognosis in persistent HPV infection in patients with HSIL. So 2-year follow-up after conization was important for improvement in clinical outcomes [16, 17]. To avoid positive margins, we found that choosing the optimal cone volume and cone depth was important. Papoutsis et al. [18] reported that in large loop excision of transformation zone (LLETZ) treatment, women with a cone volume $<$ 2.1 cm^3^ and cone depth $<$ 10 mm or a cone volume less than 8.6% of initial cervical volume were at risk of having positive margins. In contrast to cone depth, Kawano et al. [19] suggested that in women younger than 40 years, optimal cone lengths were 15 mm and 20 mm in single-quadrant and multiquadrant diseases, respectively.

Complications of conization surgery were also associated with the choice of conization modality. Marco Monti et al. [20] suggested that cervical conization had an impact on the occurrence of preterm delivery, low birth weight, and preterm premature rupture of membranes, especially in CKC and LLETZ. When CIN develops into cervical cancer, for low-risk early-stage patients, there are few differences in the results of laparoscopic or open abdominal radical hysterectomy [21].

## Conclusions

In conclusion, in patients who underwent cervical conization, the depth of conization, patient age, pathology result of conization tissue, and additional cuts influenced the positive cone margin with statistical significance. This retrospective review showed that a certain proportion of additional cuts can effectively remove the positive margins that have not been cut during conization; during conization, cutting the appropriate cone size can maintain a low positive margin rate without the need to make additional cuts, and the choice to make additional cuts is usually a doctor’s personal habit. We found that although additional cuts were effective in removing the unclear portion of the initial cone, the choice of making additional cuts often occurs in the population of doctors who use a small cone size. Therefore, we hypothesize that a suitable cone size can simultaneously avoid the appearance of positive margins and avoid the need for additional cuts. We need to evaluate more patients and develop an appropriate cone option based on different age stages and preoperative pathological results.

There is a lack of research on the use of additional cuts in cervical conization. Therefore, we still do not know whether the choice of additional cuts has an effect on the patient’s postoperative period. Unfortunately, this was not addressed in this study due to a lack of follow-up data.

## Data Availability

Not applicable.

## Abbreviations

HSIL: High-grade squamous intraepithelial lesion
LSIL: Low-grade squamous intraepithelial lesion
NILM: Negative for intraepithelial lesions or malignancy
SIL: Squamous intraepithelial lesion
HPV: Human papillomavirus
LEEP: Loop electrosurgical excision procedure
CKC: Cold knife conization
LC: Laser conization
AIS: Adenocarcinoma in situ
CIN: Cervical intraepithelial neoplasia
LLETZ: Large loop excision of transformation zone
